# Clinical and Molecular Characterizations of Mitochondrial Disorders: A Tertiary-Care Center Experience

**DOI:** 10.3390/children12081102

**Published:** 2025-08-21

**Authors:** Mohammed Almuqbil, Najla Binsabbar, Shahad Alsaif, Sulaiman Almasoud, Talah Albasry, Duaa Baarmah, Waleed Altwaijri, Ahmed Alrumayyan

**Affiliations:** 1 College of Medicine, King Saud bin Abdulaziz University for Health Sciences (KSAU-HS), Riyadh 11481, Saudi Arabia; tuwaijriw@mngha.med.sa (W.A.); rumayyana@ksau-hs.edu.sa (A.A.); 2 King Abdullah International Medical Research Center (KAIMRC), Ministry of National Guard, Riyadh 11481, Saudi Arabia; 3 Division of Pediatric Neurology, Department of Pediatrics, King Abdullah Specialist Children’s Hospital (KASCH), National Guard Health Affairs (NGHA), Riyadh 14611, Saudi Arabia; saudabinsabbarna@mngha.med.sa (N.B.); abdulazizaalsaifsh@mngha.med.sa (S.A.); dakhelaalmasoudsu@mngha.med.sa (S.A.); khaledmalbasryta@mngha.med.sa (T.A.); 4 Division of Pediatric Neurology, Department of Pediatrics, King Abdullah bin Abdulaziz Hospital (KAAUH), Princess Nourah bint Abdulrahman University (PNU), Riyadh 13412, Saudi Arabia; dmbaamah@kaauh.edu.sa

**Keywords:** disorders, genetic disorders, mitochondria, mitochondrial diseases, mitochondrial dysfunction, Saudi Arabia

## Abstract

Background: Given the limited research on mitochondrial diseases in our area, specifically regarding their genetic variability and diverse clinical manifestations, and considering the significant number of consanguineous marriages in our region, we aimed to investigate the clinical and molecular characteristics of patients with mitochondrial disorders in Saudi Arabia. Methods: This retrospective cross-sectional cohort study involved a chart review of patients diagnosed with mitochondrial disorders at the Ministry of National Guard Health Affairs tertiary health care centers in Saudi Arabia between 2013 and 2022. Results: The study population comprised 116 patients with a mean age of 10 years (±7 SD). Among the study cohort, 34.5% (*n* = 40) had died. The primary cause of death was cardiopulmonary arrest (55.0%, *n* = 22). The most prevalent condition was developmental delay (67.9%). Around 56.9% were diagnosed using Whole Exome Sequencing (WES), 10.3% by Whole Genome Sequencing due to negative WES, 23.3% through a single-gene approach, 7.8% were analyzed through a mitochondrial panel, and 1.7% via a gene panel. The distributions of current age and age at diagnosis were significantly different between the nuclear and mitochondrial gene types. Notably, the diagnostic delay time (time taken from symptom onset to genetic diagnosis) averaged 1.5 years for nDNA variants compared to an average of 10 years for mDNA variants. Conclusions: This study shows that gene type affects clinical characteristics, highlighting the importance of genetic studies in disease manifestation.

## 1. Introduction

Primary mitochondrial diseases refer to a collection of genetic abnormalities that impact mitochondria, the energy-producing organelles in human cells. These disorders hinder oxidative phosphorylation, leading to impaired energy production [1]. The range of indications and symptoms associated with mitochondrial disorders can impact any organ system, particularly those that require high levels of metabolism and energy consumption, such as the brain and liver. Mitochondria distinguish themselves from other organelles in human cells by possessing their own distinct genome. Mitochondrial homoplasmy is a term used when all mitochondrial DNAs (mtDNA) in an individual are the same. In contrast, heteroplasmy refers to a situation in which the mitochondrial DNA is divided into both normal (wild type) and mutated sub-populations. The presence of mutated sub-populations is often associated with pathogenic variants [2]. Nonetheless, mitochondrial diseases can arise from variants in either mitochondrial DNA or nuclear DNA (nDNA) [3]. Due to this dual genomic control, mitochondrial disease can be inherited in different patterns, most frequently through autosomal recessive inheritance, followed by autosomal dominant inheritance, and rarely through X-linked inheritance. The development of these patterns can be attributed to a variant in nDNA, whereas maternal inheritance is associated with variants in mtDNA due to the presence of mitochondria only in the ovum, unlike in the head of sperm [4]. However, the majority of cases are caused by spontaneous variants. Currently, approximately 400 genes have been associated with mitochondrial diseases [4], and this figure continues to grow annually due to the identification of additional de novo variants worldwide [1]. Recent research has determined that the prevalence rate of mitochondrial illness is at least 1 in every 5000 live births, making it the most prevalent ailment among inborn abnormalities of metabolism [4]. According to the Centre Arab Genomic Studies, a total of 682 genetic disorders, including mitochondrial diseases, were found, along with 575 known gene alterations in Saudi Arabia and other Gulf nations, of which Saudi Arabia was responsible for 49% [5]. The most frequently reported conditions include Leigh syndrome, Leber Optic Atrophy, and Mitochondrial Myopathy, Encephalopathy, Lactic Acidosis, and Stroke Like Episodes (MELAS) [5]. Prior to the development of genetic research, medical professionals identified patients with mitochondrial disorders based on their clinical observations. Advancement in genetic analysis have revealed that mitochondrial disorders are not caused by a single genetic factor but rather by a wide range of genetic variations, resulting in diverse and complex symptoms [5,6,7,8,9,10,11,12,13].

Previous studies in Saudi Arabia have examined mitochondrial disorders among various groups of patients, including those with diabetes mellitus and multiple sclerosis [14,15]. There is limited research on mitochondrial diseases in Saudi Arabia, specifically regarding their genetic variability and diverse clinical manifestations, considering the significant number of consanguineous marriages in our region (with a prevalence rate ranging between 20% and 50%) [16]. This is expected to contribute to a higher prevalence of recessive mitochondrial disorders due to the higher probability of inheriting recessive genetic variants within closely related populations. Therefore, we conducted a study to examine the primary genetic factors associated with mitochondrial disorders and their corresponding clinical presentations. The aim of this study is to investigate the clinical and molecular characteristics of patients with mitochondrial disorders in Saudi Arabia. Specifically, we aimed to delve into the key genetic variables linked to mitochondrial disorders in our patients. Specific objectives include investigating the primary genetic variables in patients diagnosed with mitochondrial disorders using genetic testing results (whole exome, whole genome, and mitochondrial genetic studies), and analyzing the clinical presentations observed in these patients. This is important for achieving an accurate diagnosis and, hence, personalized treatment by understanding the disease mechanisms for potential therapies, which guide appropriate medical interventions.

## 2. Materials and Methods

### 2.1. Study Design

This retrospective cross-sectional cohort study involved a chart review of patients diagnosed with mitochondrial disorders at the Ministry of National Guard Health Affairs tertiary health care centers in Saudi Arabia between 2013 and 2022.

### 2.2. Study Population and Settings

All patients diagnosed with a confirmed molecular diagnosis of a mitochondrial disorder with mutated mDNA or nDNA, specifically those with pathogenic variants, were included in this study. Diagnosis was confirmed by identifying a gene that causes a mitochondrial disorder and matches the patient’s phenotype. Patients without a certain diagnosis that correlated with mitochondrial disease (negative genetic test results or variants of unknown significance), patients with a non-mitochondrial diagnosis, or insufficient medical reports were excluded.

### 2.3. Data Collection

Data were collected by expert healthcare professionals in the field of neurology to verify the patients’ diagnoses. Data collection process involved chart review and data extraction from patients’ electronic medical records. Data collection involved a comprehensive review of patient charts using a detailed data collection sheet designed by the research team. This study collected a variety of clinical, seizure-related, and genetic characteristics. Clinical characteristics included the diagnosis and prevalence of developmental delays (global, language, motor, and social), endocrinopathies (poor growth, short stature, hypoglycemia, adrenal insufficiency, diabetes mellitus, growth hormone deficiency, hypoparathyroidism, hypothyroidism, pancreatic insufficiency, hypogonadism, and syndrome of inappropriate anti-diuretic hormone release), epilepsy, spasticity, cardiomyopathy, optic atrophy, encephalopathy, myopathy, ophthalmoplegia, ataxia, chorea, sensorineural deafness, exercise intolerance, ptosis, migraine, stroke-like episodes, and pigmentary retinopathy. Seizure-related characteristics included the types of seizures (generalized tonic-clonic, focal motor clonic, focal motor tonic, myoclonic, infantile spasms, and focal to bilateral tonic-clonic) and the use of anti-seizure medications (multiple or single medications). Genetic characteristics encompassed the type of genetic tests performed, including whole-exome sequencing (WES), single-gene tests, whole-genome sequencing (WGS), mitochondrial panel, and gene panel.

### 2.4. Ethical Approval

Institutional Review Board approval was obtained from the King Abdullah International Medical Research Center (KAIMRC) with approval number NRC22R/040/01. AI was not used for writing or editing the manuscript text. Written informed consent was obtained from all participants prior to genetic testing, including explicit permission for data use in publications and international data repositories. For minors, assent was obtained in addition to the guardian’s consent. All samples and associated data were anonymized to ensure participant confidentiality. Genetic counseling was offered to participants before and after testing in accordance with ethical guidelines.

### 2.5. Statistical Analysis

The data for this study were analyzed using the Statistical Package for the Social Sciences Software (SPSS), version 29.0.1, from IBM Corp., Armonk, NY, USA. Continuous variables are presented as mean and standard deviation. The chi-squared test and Fisher’s Exact Test were used to examine the differences in gene type distribution across different patient characteristics and outcomes. Multiple logistic regression analyses were conducted to identify the predictors of mitochondrial gene type and patient survival. The findings of the logistic regression are presented as adjusted odds ratios with 95% confidence intervals. The significant value of *p* was set to 0.05.

## 3. Results

### 3.1. Patients’ Demographic and Clinical Background Characteristics

**Figure 1:**
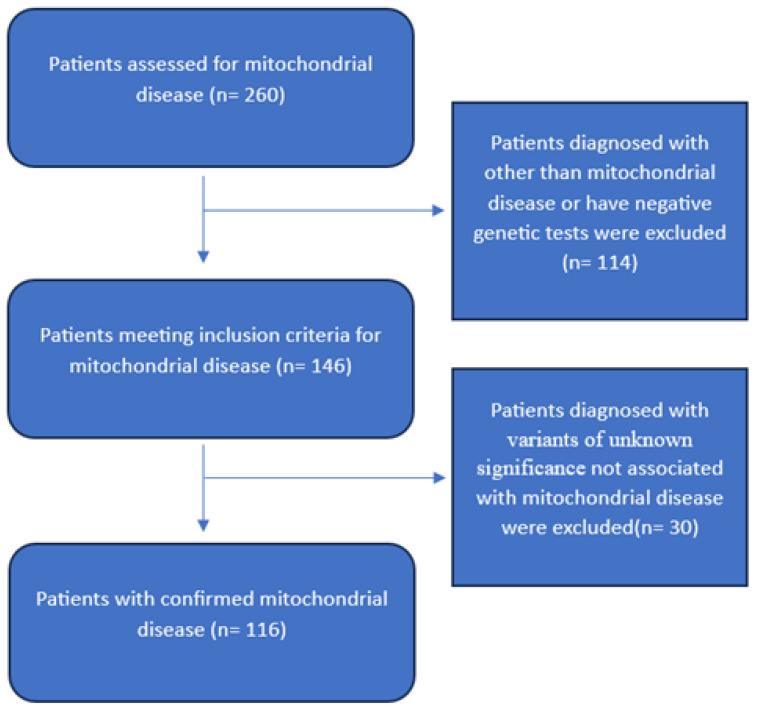
Study sample selection flowchart.

A total of 260 patients were recruited, of whom 114 were excluded due to diagnoses other than mitochondrial disease, negative molecular testing, or variants of unknown significance not matching mitochondrial diseases, as shown in Figure 1.

The study population comprised 116 patients, with a sex distribution of 54.3% female (*n* = 63) and 45.7% male (*n* = 53). The current age of the patients ranged from 1 to 59 years, with a mean age of 10 years (±7 SD). The age of onset for mitochondrial disorders varied from 0 to 52 years, with an average of 2 years (±5.6 SD). Patients were diagnosed at an average age of 5.3 years (±8 SD), with the age at diagnosis spanning from 1 to 59 years. Among the study cohort, 34.5% (*n* = 40) had died, with an average age at death of 3 years (±4 SD), ranging from 0 to 17.6 years. The primary causes of death included cardiopulmonary arrest (55.0%, *n* = 22), respiratory failure (17.5%, *n* = 7), and other causes (27.5%, *n* = 11), including liver failure (*n* = 3), heart failure (*n* = 2), septic shock (*n* = 2), hypovolemic shock (*n* = 1), sudden unexpected death in epilepsy (*n* = 1), metabolic crisis (*n* = 1), and brain death (*n* = 1). Additionally, a significant proportion of patients had a family history of mitochondrial disease (59.5%, *n* = 69), and consanguinity was reported in 81.9% (*n* = 95) of the cases. For further details on patients’ demographic and clinical background characteristics, refer to Table 1. The regional distribution of cases is shown in Supplemental Figure S1.

Figure S2 in the Supplementary Material shows the clinical characteristics of the patients. The most prevalent condition was developmental delay (67.9%), of which the most common subtype was global developmental delay (88.8%), followed by language delay (6.3%), motor delay (3.8%), and social delay (1.3%). The second most frequent clinical finding was endocrinopathy in 34.5% of patients, with poor growth representing 19.8% of those, followed by short stature in 12.9%, and the rest had different endocrinological abnormalities, as detailed in Table 1.

Epilepsy was observed in 28.4% of patients, of which generalized tonic-clonic seizures accounted for 12.9%, followed by focal motor clonic seizures in 9.5%. Most patients with epilepsy have drug-resistant epilepsy, as reflected by the use of multiple anti-seizure medications, as seen in 18.1% vs. 9.5% on a single anti-seizure medication (Table 2).

Regarding the genetic characteristics of the study participants, 56.9% were diagnosed using whole-exome sequencing (WES), 10.3% using whole-genome sequencing (WGS) due to negative WES, 23.3% using a single-gene approach, 7.8% using a mitochondrial panel, and 1.7% using a gene panel. A total of 27 patients were tested using single-gene testing, and that was due to a family history of the same clinical presentation, where a mitochondrial gene was already found. The 27 genes tested in this way were AAAS in 8 patients, ISCA2 in 5 patients, POLG in 2 patients, SLC25A42 in 2 patients, SURF1 in 2 patients, and ACAT1, DGUOK, ELAC2, LRPPRC, NDUFS1, PARS2, RARS2, and RAB3GAP2 in a single patient each. Mitochondrial sequencing was integrated into either WGS or WES, except for nine patients (7.8%) who were diagnosed based on the mitochondrial panel, in which mitochondrial DNA sequencing was performed separately. The resulting syndromes varied, with the most common being multiple mitochondrial dysfunction syndrome 4 (ISCA2) in 13.8%, achalasia addisonian alacrima syndrome (AAAS) in 8.6%, and mitochondrial complex I, III, V, or IV syndromes (TMEM70, MT-ND5, and ATP5MD, among others) in 12.9%. The gene type was mostly nuclear, as seen in 93.1%, and the remaining was mitochondrial in 6.9%. Of these, the variant class was class I (pathogenic) in 68.1% and class II (likely pathogenic) in 17.2%. The genetic characteristics are summarized in Table 3 and Supplemental Figure S3.

Regarding neuroradiological investigations, 50% of patients had abnormal brain MRI results, with white matter changes in 31.9%, basal ganglia changes in 16.4%, cerebellar changes in 6.9%, brain malformation in 6%, and mixed changes in 5.2%. Regarding the MRS results, 14.7% were abnormal.

### 3.2. Gene Type Stratified by Patients’ Demographic Characteristics

Table 4 presents the gene types stratified by the patients’ demographic characteristics. There were no statistically significant differences in terms of patients’ sex, mortality, cause of death, family history of mitochondrial disease, and consanguinity (*p* > 0.05).

For current age and age at diagnosis, each compared to the type of gene, whether nuclear or mitochondrial, there was a statistically significant difference with a *p*-value of less than 0.001, indicating that the distribution of ages was significantly different between the nuclear and mitochondrial gene types. Notably, the diagnostic delay time (time taken from symptom onset to genetic diagnosis) averaged 1.5 years for nDNA variants compared to an average of 10 years for mDNA variants (Table 4).

In terms of clinical characteristics, there was a statistically significant difference in poor growth based on gene type, where it was observed in 19 out of 23 cases (82.6%) with the nuclear gene type, while for the mitochondrial gene type, 4 out of 23 cases (17.4%) showed poor growth (*p* = 0.048). Another significant difference emerged based on seizure type and gene type, as focal motor clonic seizure type was reported in 8 out of 11 cases (72.7%) with nuclear gene type, whereas 3 of the 11 cases (27.3%) with mitochondrial gene type exhibited this seizure pattern (*p* = 0.028). Additionally, five of eight cases (62.5%) with the nuclear gene type had ataxia, compared to three of eight cases (37.5%) with the mitochondrial gene type (*p* = 0.011). However, other clinical characteristics did not differ significantly based on gene type (*p* > 0.05). For further details concerning pathogenic and likely pathogenic variants, including HGVS nomenclature, zygosity, and ACMG classification, see Supplementary Table S1.

Table 5 presents the patients’ basic metabolic workup. The most commonly identified abnormality in patients’ metabolic workup was increased lactic acid levels (50.0%).

### 3.3. Factors Associated with Having Mitochondrial Gene Type

Multiple logistic regression analysis, adjusting for age and consanguinity, did not identify any statistically significant variables associated with the mitochondrial gene type (*p* > 0.05) (Table 6).

### 3.4. Factors Associated with Survival

Multiple logistic regression analysis identified that patients with a family history of mitochondrial disease were less likely to survive, with an odds ratio of 0.18 (95% CI: 0.04–0.87) (*p*-value: 0.030) (Table 7).

## 4. Discussion

The results of our study indicate that the average age of symptom onset is 2 ± 5.6 years, whereas the average age at which a diagnosis is made is 5.3 ± 8 years. This suggests that there is often a significant delay between the onset of symptoms and confirmation of diagnosis. These findings confirm the observations of Paiva et al. [17]. In addition, the average age at death was 3 ± 4.1 years, suggesting significant variation in mortality rates. This is consistent with the findings of Keshavan et al.’s comprehensive analysis, which reported that 74% of their study group died within a span of less than 10 years [18].

The study population exhibited a notable prevalence of cardiopulmonary arrest as the primary cause of death. This highlights a significant health issue in this population, which is consistent with the findings of Elliott et al. They concluded that patients with mitochondrial disease have an overall heightened risk of cardiovascular morbidity and mortality [19]. Furthermore, the study results demonstrated a significant prevalence of familial history of mitochondrial disease in 59.5% of patients and consanguinity in 81.9% of cases. These findings are consistent with those of Algazali et al., who reported a high frequency of consanguinity in Saudi patients diagnosed with genetic disorders, with an average rate ranging from 42% to 67% across the entire Arab world [20]. The prevalence of epilepsy in 28.4% of cases indicates that it is a rather common condition. This finding aligns with that of Whittaker et al., who also found a comparable rate of epilepsy in a group of individuals with mitochondrial diseases [21]. Furthermore, the utilization of several anti-seizure medications, observed in 18.1% of cases, as opposed to the use of a single anti-seizure medication in 9.5% of cases, highlights the difficulty in managing epilepsy in this particular group. These findings are consistent with those of Lopriore et al. [22]. Furthermore, a significant 88.8% of the participants had global developmental delay, underscoring the considerable influence of this disease on neurodevelopment. This aligns with the findings of Falk et al., who observed a comparable pattern in their study on mitochondrial diseases [23]. In addition, the higher prevalence of nuclear genes (93.1%) than mitochondrial genes (6.9%) highlights the complex nature of mitochondrial diseases, as observed in the study by Angelini et al. [24].

Basic metabolic workup revealed probable biochemical anomalies linked to the disease. Significantly, 33.6% of cases demonstrated abnormal ammonia levels, suggesting that a significant proportion of the population may have problems related to ammonia metabolism. This is supported by the research of Niknahad et al., who also found a comparable prevalence of high ammonia levels in patients with mitochondrial disease [25]. Metabolic acidosis, a condition characterized by elevated amounts of lactic acid, is commonly found in 50.0% of cases, as observed by Andersen et al. in patients with mitochondrial disease [26]. Our study found that 50% of patients who received brain imaging had abnormal MRI results, which aligns with prior research conducted by Saneto et al. They also identified a considerable prevalence of abnormal MRI findings in patients with mitochondrial illnesses [27]. Particular deviations, such as alterations in white matter observed in 31.9% of cases and changes in the basal ganglia observed in 16.4% of cases, correspond to the typical neuroimaging patterns observed in mitochondrial diseases, as highlighted by Gropman et al. [28]. The MRS, EEG, and EMG/NCS results highlighted the wide range of neurological symptoms observed in this group. The significant occurrence of aberrant EEG backgrounds, mostly defined by slowing, was observed in 91.4% of cases, indicating the elevated frequency of encephalopathy in individuals with mitochondrial disease, as reported by Chevallier et al. [29]. Moreover, the atypical EMG/NCS findings suggest that 75% of the cases include damage to the axons, whereas 25% involve damage to the myelin sheath. These results align with the peripheral neuropathy commonly observed in individuals with mitochondrial diseases, as described in the study conducted by Horvath et al. [30]. No statistically significant differences were found in the patients’ sex, age at onset, diagnosis, or death, or the types of nuclear and mitochondrial genes. These findings differ to some extent from those of Ng et al., who indicated a minor preference for sex in relation to mitochondrial gene involvement [31]. Nevertheless, the absence of substantial disparities in this study indicates that sociodemographic factors may have a limited impact on the specific genes associated with mitochondrial disease in this particular group.

Our investigation revealed considerable disparities in the present age and age at formal diagnosis based on the type of gene, whether nuclear or mitochondrial. This suggests that there is a clear age distribution dependent on the type of gene, which could indicate differences in when and how a disease develops. These results are consistent with the findings of Chistiakov et al., who similarly observed notable age-related disparities in mitochondrial diseases associated with mitochondrial and nuclear genes [32]. In contrast, there was no substantial disparity between the age at symptom onset and the age at death among deceased patients. Therefore, in this group, these factors were not closely linked to the specific genes involved. There was a statistically significant difference in the prevalence of poor growth based on gene type, with a higher prevalence rate of poor growth in cases where nuclear genes were involved (82.6%) than in cases in which mitochondrial genes were involved (17.4%). This finding is consistent with the research conducted by Goldstein et al. (2013), who found that growth retardation is a frequent symptom of mitochondrial diseases associated with nuclear genes [33]. Furthermore, a notable disparity was observed in the prevalence of focal motor clonic seizures among different genetic types. The incidence rate of nuclear genes was higher (72.7%) than that of mitochondrial genes (27.3%). This finding aligns with the discoveries of Wesół-Kucharska et al. (2021), who emphasized a comparable pattern in their study of seizure patterns in individuals with mitochondrial disease [34]. Similarly, the variation in the prevalence of ataxia based on genetic type corresponds to the existing body of research on mitochondrial diseases. Mitochondrial gene involvement is frequently linked to ataxia, as evidenced by Lopriore et al. [35]. It is crucial to acknowledge that although these disparities are statistically significant, mitochondrial diseases exhibit a high level of heterogeneity, resulting in a wide range of clinical presentations. Hence, personalized evaluation and treatment remain essential in the setting of mitochondrial diseases.

Accurate and early diagnosis by identifying specific genetic mutations common in the Saudi population will enhance targeted treatment strategies that can be performed in relation to the prevailing genetic profile. In addition, the establishment of a genetic and clinical database on Saudi patients with mitochondrial disorders will allow effective genetic counseling and improvement in risk assessment within families to inform health policy on prevention strategies. This includes pre-marital genetic counselling for high-risk populations known to have pathogenic variants. In clinical practice, this could involve screening infants for specific neuromuscular and developmental abnormalities and potential genetic disorders. This will enhance early detection and management, which will ultimately improve patient outcomes.

One limitation of this study is that it relies on retrospective data, which can result in missing or biased information. In addition, the limited sample size may restrict the capacity to apply the findings to a larger population. The absence of comprehensive data on specific variables, such as the accessibility of information on particular clinical and genetic characteristics, may have impacted the thoroughness of the analysis. Sequencing depth, mtDNA coverage, and details of the bioinformatic pipelines were not uniformly available in this study, as testing was performed across multiple external laboratories in a retrospective manner. Future prospective studies with centralized sequencing and analysis will allow for more standardized reporting. Moreover, the study’s observational design hinders the ability to establish cause-and-effect associations, and the presence of confounding factors may have impacted certain associations.

In conclusion, this research offers a comprehensive overview of individuals with mitochondrial diseases. This study identified significant differences in clinical characteristics based on gene type, emphasizing the relevance of genetic analysis in understanding disease manifestations. While limitations exist, these findings contribute to the knowledge base surrounding mitochondrial diseases, potentially guiding future research and clinical management strategies for affected individuals, as no previous similar studies have been conducted for this study population. However, further investigations with larger, more diverse samples (multi-centre studies that involve comparisons with different countries) and prospective designs may help confirm and expand the insights gained from this study.

## Figures and Tables

**Table 1:** Patients’ demographic and clinical background characteristics.

Variable	Frequency (Percentage)	
Sex		
Female	63 (54.3%)	
Male	53 (45.7%)	
	Mean ± SD	Range
Current age (Years)	10 ± 7	1–59
Age of Onset (Years)	2 ± 5.6	0–52
Age at Diagnosis (Years)	5.3 ± 8	1–59
Age at Death (Years)	3 ± 4	0–17.6
Mortality		
Yes	40 (34.5%)	
No	76 (65.5%)	
Cause of Death		
Cardiopulmonary arrest	22 (55.0%)	
Respiratory Failure Other	7 (17.5%) 11 (27.5%)	
Family history of mitochondrial disease	69 (59.5%)	
Consanguinity	95 (81.9%)	
Clinical feature		
Developmental delay	79 (67.9%)	
Global	70	
Language	5	
Motor	3	
Social	1	
Endocrinopathy	40 (34.5%)	
Poor growth	23	
Short stature	15	
Hypoglycemia	9	
Adrenal insufficiency	9	
Diabetes mellitus	2	
Growth hormone deficiency	2	
Hypoparathyroidism	1	
Hypothyroidism	1	
Pancreatic insufficiency	1	
Hypogonadism	1	
Syndrome of Inappropriate Anti-Diuretic Hormone Release	1	
Epilepsy	33 (28.4%)	
Spasticity	32 (27.8%)	
Cardiomyopathy	25 (21.6%)	
Optic atrophy	18 (15.5%)	
Encephalopathy	18 (15.5%)	
Myopathy	13 (11.2%)	
Ophthalmoplegia	9 (7.8%)	
Ataxia	8 (7.0%)	
Chorea	8 (7.0%)	
Sensorineural deafness	8 (7.0%)	
Exercise intolerance	6 (5.2%)	
Ptosis	5 (4.3%)	
Migraine	3 (2.6%)	
Stroke-like episodes	2 (1.7%)	
Pigmentary retinopathy	1 (0.9%)	

**Table 2:** Seizure-related characteristics.

	Frequency (Percentage)
Seizure Type	
Generalized tonic clonic	15 (12.9%)
Focal, motor, clonic	11 (9.5%)
Focal, motor, tonic	5 (4.3%)
Myoclonic	3 (2.6%)
Infantile spasms	3 (2.6%)
Focal to bilateral tonic clonic	2 (1.7%)
Anti-seizure Medications	
Multiple	21 (18.1%)
Single	11 (9.5%)

**Table 3:** Genetic Characteristics.

	Frequency (Percentage)
Type of test	
Whole Exome Sequencing (WES)	66 (56.9%)
Single Gene	27 (23.3%)
Whole Genome Sequencing (WGS)	12 (10.3%)
Mitochondrial Panel	9 (7.8%)
Gene Panel	2 (1.7%)

**Table 4:** Gene type stratified by patients’ demographic characteristics.

Demographic Characteristics	Type of Gene		*p*-Value
	Nuclear	Mitochondrial	
	Frequency Percentage	Frequency (Percentage)	
Sex			
Male	50 (94.3%)	3 (5.7%)	0.725
Female	58 (92.1%)	5 (7.9%)	
Mortality			
Yes	39 (97.5%)	1 (2.5%)	0.260
No	69 (90.8%)	7 (9.2%)	
Cause of Death			
Respiratory Failure	7 (100.0%)	0 (0.0%)	0.450
Cardiopulmonary arrest due to an unknown cause	22 (100.0%)	0 (0.0%)	
Others	10 (90.9%)	1 (9.1%)	
Family history of mitochondrial disease			
Yes	66 (95.7%)	3 (4.3%)	0.266
No	42 (89.4%)	5 (10.6%)	
Consanguinity			
Yes	88 (92.6%)	7 (7.4%)	1.000
No	20 (95.2%)	1 (4.8%)	
Median age differences stratified by gene type			
	Median (IQR)	Median (IQR)	*p*-Value
Current age (Years)	8 (4–13)	17 (15–22)	<0.001 *
Age of Onset (Years)	0.5 (0–1)	1.5 (0–10)	0.675
Age at Diagnosis (Years)	2 (0.8–6)	11.5 (7–16)	<0.001 *
Age at Death (Years)	1.3 (0.8–3)	17.6 (17.6–17.6)	0.091

* *p*-value less than 0.05.

**Table 5:** Basic Metabolic Workup.

		Frequency (Percentage)
Ammonia		
Normal		55 (47.4%)
Abnormal		39 (33.6%)
Not Available		22 (19.0%)
Lactic Acid		
Normal		44 (37.9%)
Increased		58 (50.0%)
Not Available		14 (12.1%)
Thyroid-Stimulating Hormone		
Normal		55 (47.4%)
High		9 (7.8%)
Low		3 (2.6%)
Not Available		49 (42.2%)
T4		
Normal		43 (37.1%)
High		1 (0.9%)
Low		5 (4.3%)
Not Available		67 (57.8%)
Urine Organic Acids		
Normal		35 (30.2%)
Abnormal		14 (12.1%)
Not Available		67(57.8%)
Plasma Amino Acids		
Normal		33 (28.4%)
Abnormal		
Not Available		

**Table 6:** Factors associated with mitochondrial gene type.

Variable	Adjusted Odds Ratio of Having Mitochondrial Gene Type 95% Confidence Interval ^§^	*p*-Value
Sex		
Male (Reference category)	1.00	
Female	1.63 (0.33–8.06)	0.550
Mortality		
No (Reference category)	1.00	
Yes	0.39 (0.04–3.70)	0.411
Epilepsy		
No (Reference category)	1.00	
Yes	0.98 (0.16–5.94)	0.981
Family history of mitochondrial disease		
No (Reference category)	1.00	
Yes	0.42 (0.09–2.01)	0.275

^§^ Adjusted for confoudning variables.

**Table 7:** Factors associated with survival.

Variable	Adjusted Odds Ratio for Patients’ Survival 95% Confidence Interval	*p*-Value
Sex		
Male (Reference category)	1.00	
Female	1.01 (0.19–5.40)	0.996
Consanguinity		
No (Reference category)	1.00	
Yes	0.77 (0.09–6.96)	0.815
Family history of mitochondrial disease		
No (Reference category)	1.00	
Yes	0.18 (0.04–0.87)	0.033 *
Anti-seizure medication patterns		
Single anti-seizure medication (Reference category)	1.00	
Muliple anti-seizure medication	1.19 (0.27–5.29)	0.821

* *p* < 0.05.

## Data Availability

The original contributions presented in this study are included in the article. Further inquiries can be directed to the corresponding author.

## Supplementary Materials

The following supporting information can be downloaded at: https://www.mdpi.com/article/10.3390/children12081102/s1, Figure S1: Regional distribution of the cases; Figure S2: Clinical Characteristics; Figure S3: Genes identified; Figure S4: Brain MRI of a 9-year-old boy diagnosed with an ISCA2 gene mutation causing Multiple Mitochondrial Dysfunction syndromes; Figure S5: Longitudinal bipolar montage of an EEG for a 12-year-old girl with a PDHA1 gene mutation causing pyruvate dehydrogenase deficiency, showing sharp-slow waves in the right fronto-central head region; Figure S6: EEG in reference cz montage for a 10-year-old girl with NDUFS1 gene mutation causing mitochondrial complex 1 deficiency showing abundant bi-temporal sharp and slow wave complexes, more on the left head side; Figure S7: Longitudinal bipolar montage of an EEG for a 15-year-old girl with a FASTKD2 gene mutation causing combined oxidative phosphorylation type 44, showing intermittent semi-rhythmic delta slowing intermixed with low amplitude epileptic discharge over the left frontal region; Table S1: Pathogenic Variants.
