# Supplementary material for: Clinical and Molecular Characterizations of Mitochondrial Disorders: A Tertiary-Care Center Experience
Source: Children (Basel). 2025 Aug 21;12(8):1102. doi: 10.3390/children12081102 (PMC12384868; doi:10.3390/children12081102)
Supplement: Supplementary file 1 [file children-12-01102-s001.zip › children-3727203-supplementary.pdf]

Supplemental Figures

Figure S1. Regional distribution of the cases

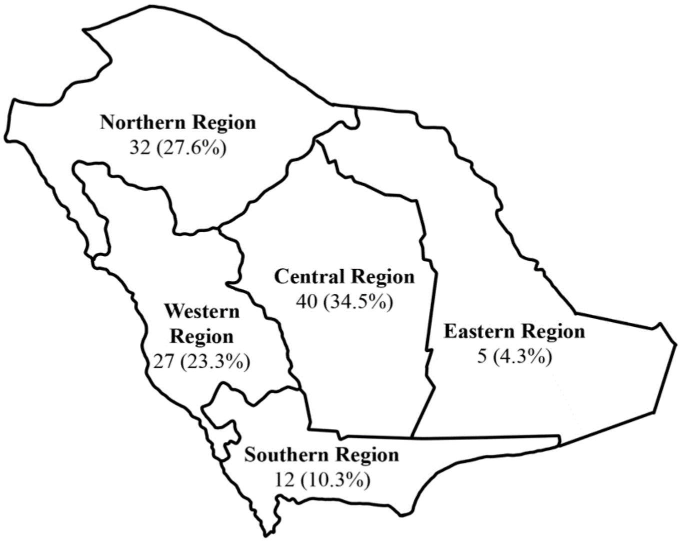

Figure S2. Clinical Characteristics

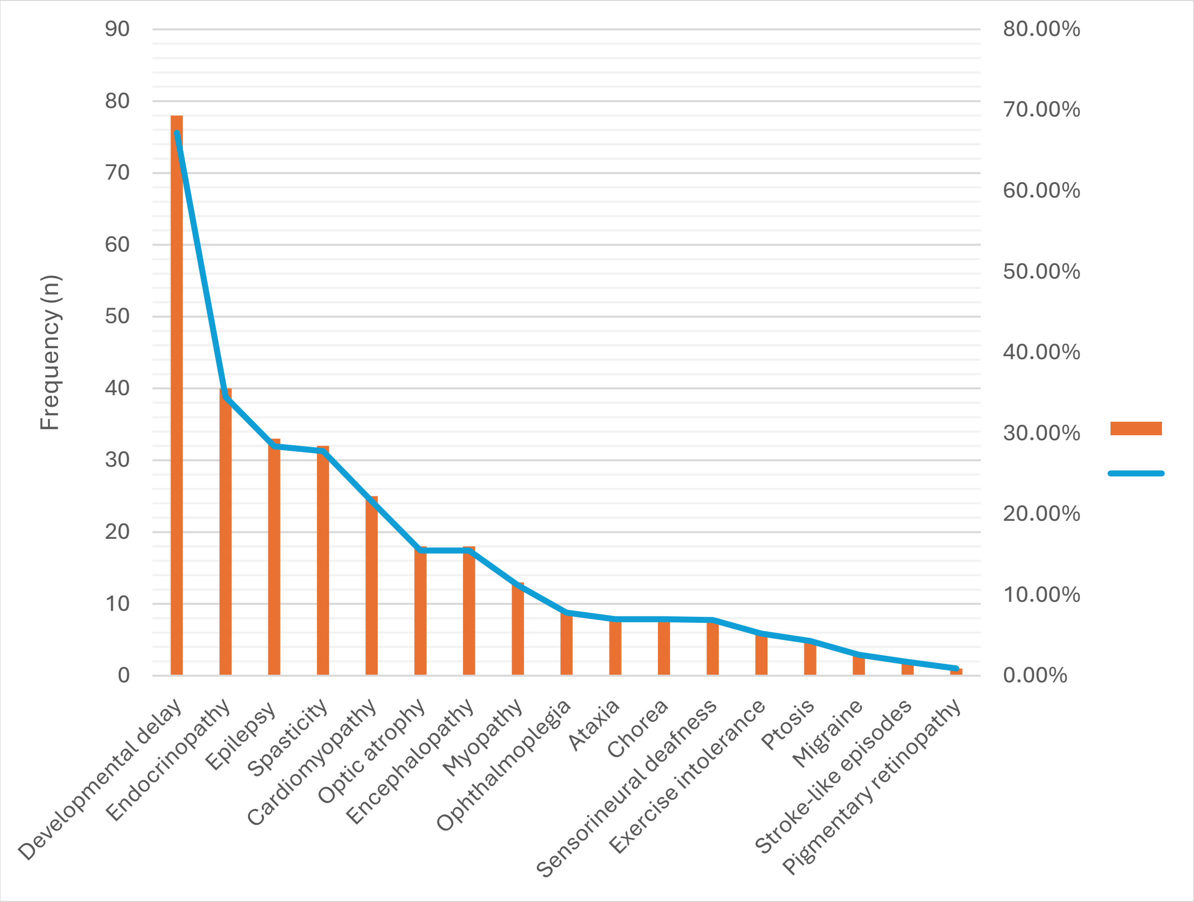

Figure S3. Genes identified.

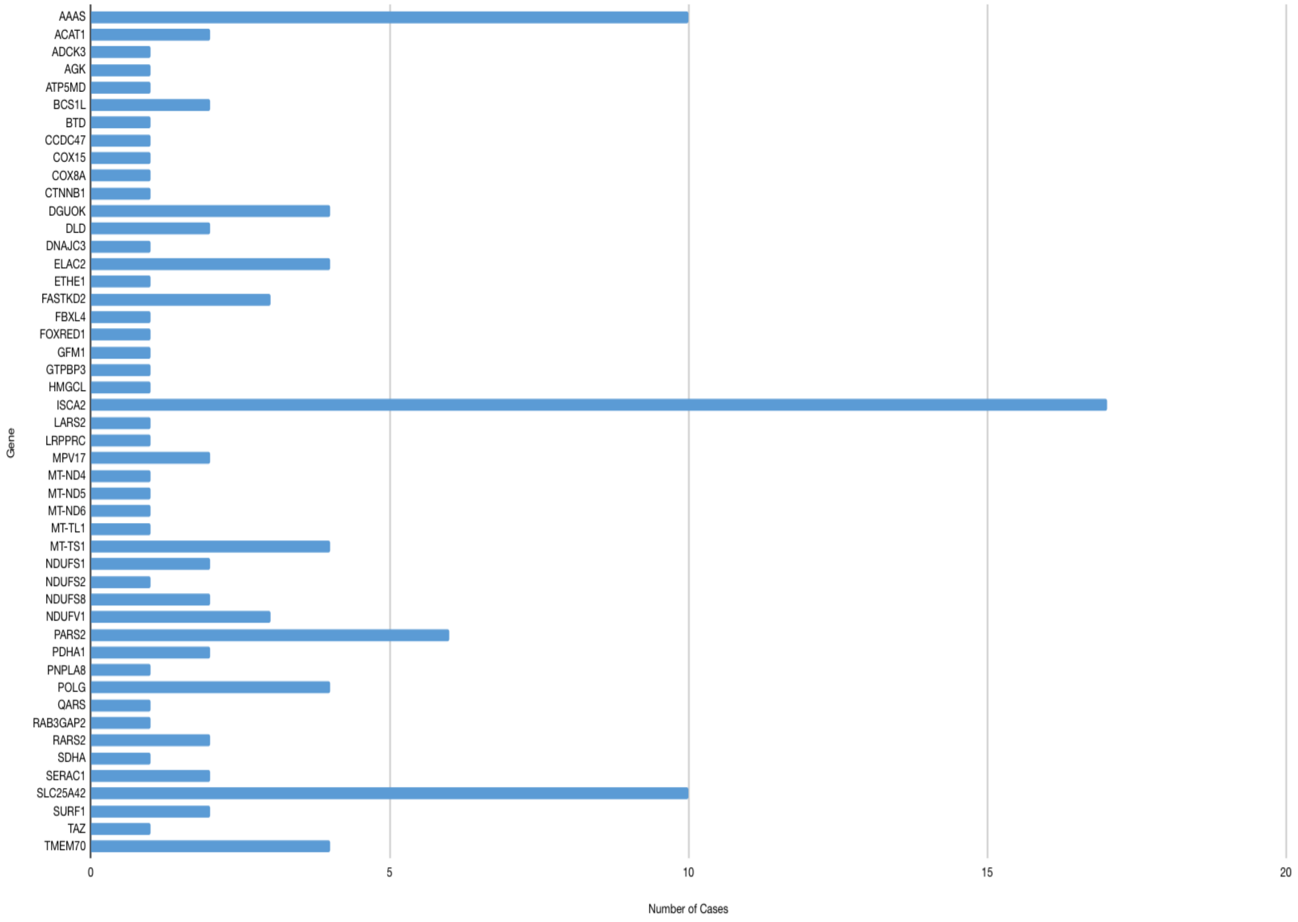

Figure S4. Brain MRI of a 9-year-old boy diagnosed with an ISCA2 gene mutation causing Multiple Mitochondrial Dysfunction syndromes. MRI performed at the age of 2 years, showing in an axial T2 FLAIR: diffuse, homogeneous abnormal signal involving the supratentorial superficial and deep white matter (A) with unremarkable basal ganglia and partial involvement of the left posterior limb of the internal capsule and partial involvement of the splenium of the corpus callosum. There is also involvement of the cerebellar deep white matter (C) up to the inferior cerebellar peduncle (E). The brainstem shows partial medullary involvement with an abnormal signal involving the cervico-medullary portion of the spinal cord (G). The above-described areas showed restricted diffusion (B,D,F, and H).

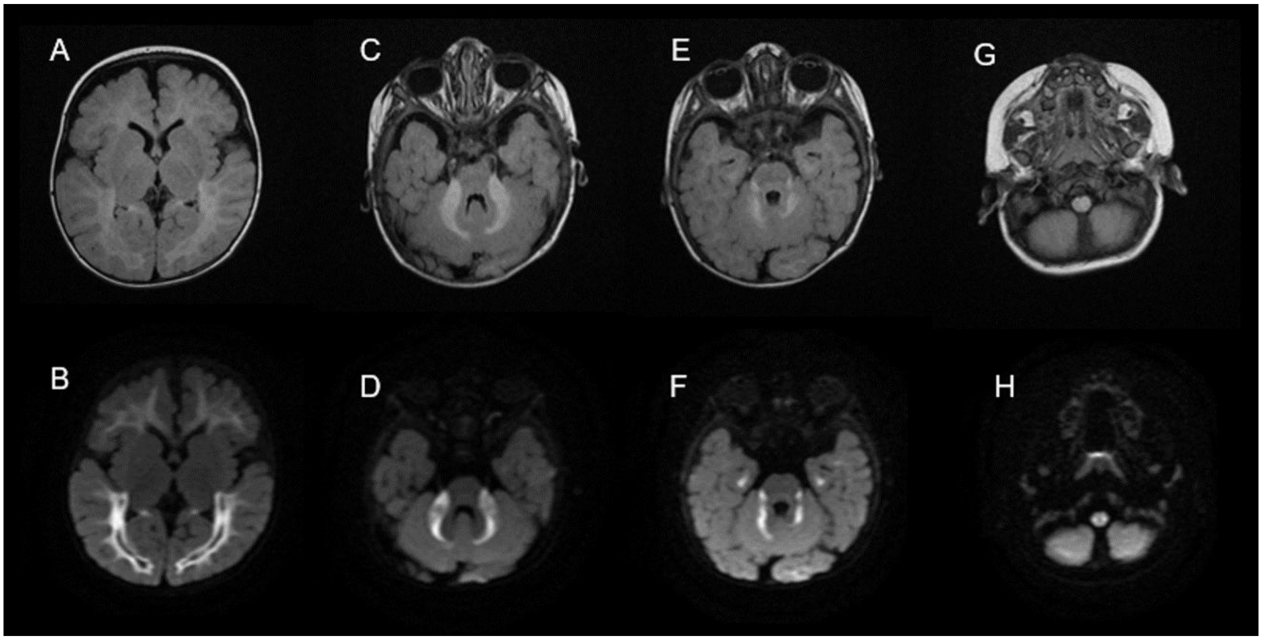

Figure S5. Longitudinal bipolar montage of an EEG for a 12-year-old girl with a PDHA1 gene mutation causing pyruvate dehydrogenase deficiency, showing sharp-slow waves in the right fronto-central head region.

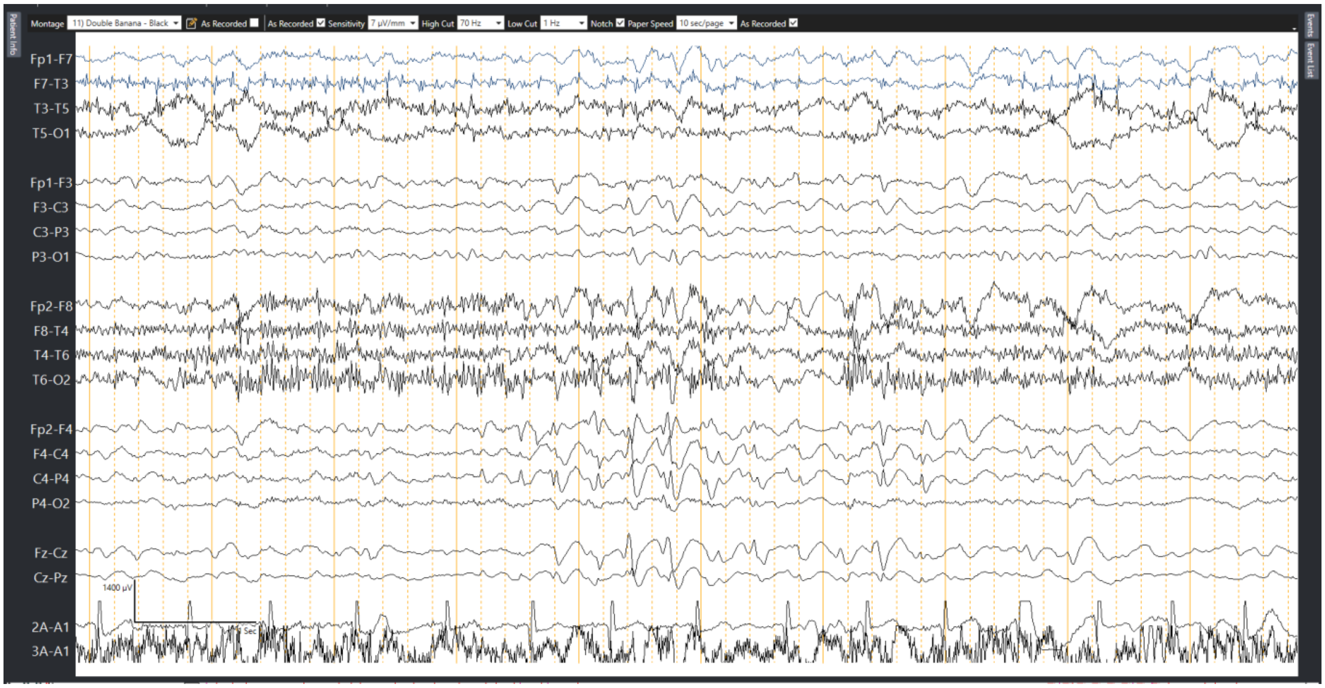

Figure S6. EEG in reference cz montage for a 10-year-old girl with NDUFS1 gene mutation causing mitochondrial complex 1 deficiency showing abundant bi-temporal sharp and slow wave complexes, more on the left head side.

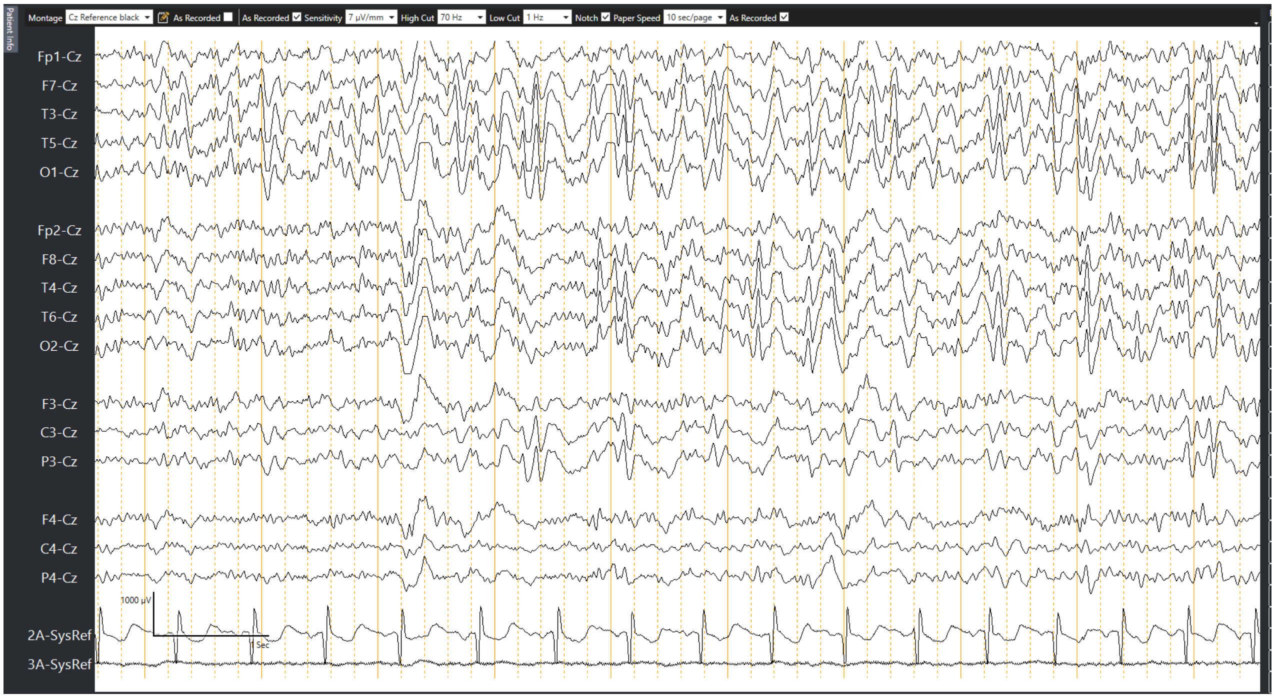

Figure S7. Longitudinal bipolar montage of an EEG for a 15-year-old girl with a FASTKD2 gene mutation causing combined oxidative phosphorylation type 44, showing intermittent semi-rhythmic delta slowing intermixed with low amplitude epileptic discharge over the left frontal region.

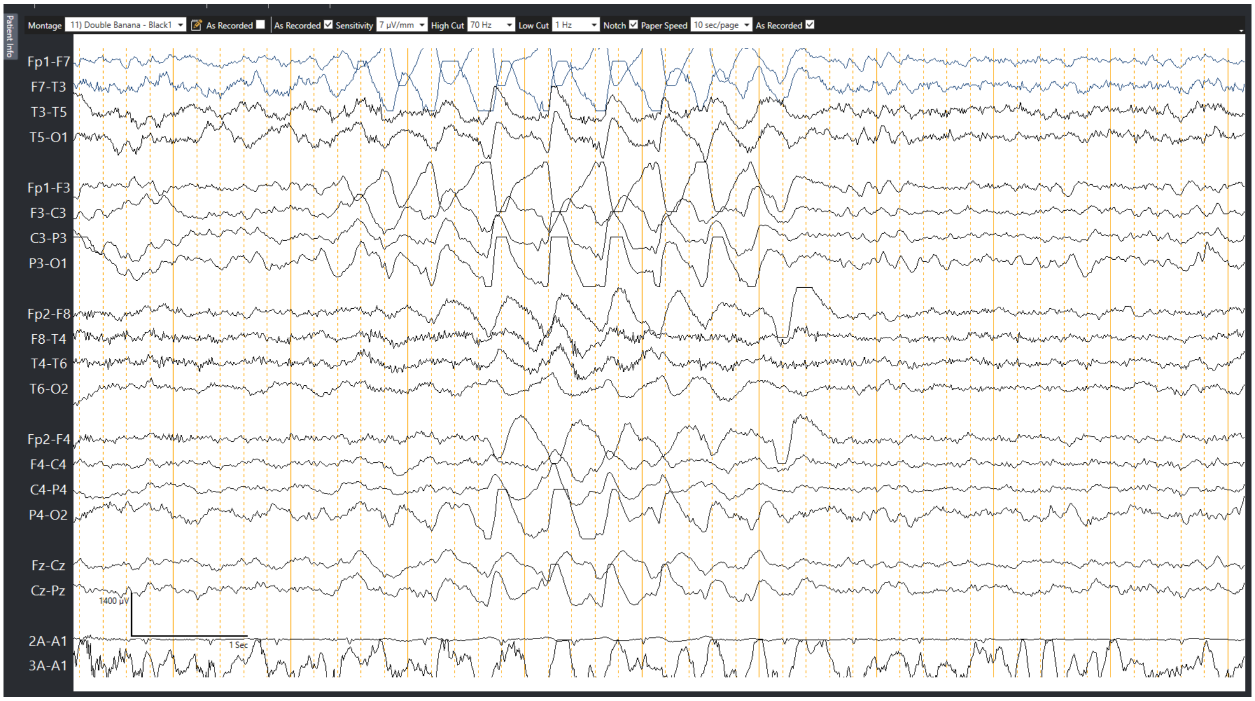

Supplementary Table S1. Pathogenic Variants

Gene	HGVS Nomenclature	Zygosity	ACMG Classification
AAAS	c.43C>A	Homozygous	P
AAAS	c.43C>A	Homozygous	P
AAAS	c.43C>A	Homozygous	P
AAAS	c.1432C>T	Homozygous	P
AAAS	c.1432C>T	Homozygous	P
AAAS	c.1432C>T	Homozygous	P
AAAS	c.1432C>T	Homozygous	P

AAAS	c.1432C>T	Homozygous	P
AAAS	c.1432C>T	homozygous	P
ACAT1	c.410_418delinsT	Homozygous	LP
ACAT1	c.410_41delinsT	homozygous	LP
ADCK3	c.814 G>A	homozygous	LP
AGK	c.424-3C>G	Homozygous	P
ATP5MD	c.59A>G	Homozygous	VUS
BCS1L	c.385G>A	Homozygous	P
BCS1L	c.441 C>T	homozygous	LP
BTD	c.1336 G>C	homozygous	P
CCDC47	c.567_570del	Homozygous	P
COX15	c.649C>T	Homozygous	P
CTNNB1	c.997dup	Heterozygous	P
DGUOK	c.737C>T	Compound Heterozygous	P
DGUOK	c.140T>A	Homozygous	VUS
DGUOK	c. 766 dup	homozygous	P
DGUOK	c.763_dup	homozygous	P
DLD	c.685G>T/c.684+1G>T	Compound Heterozygous	P
DLD	c.685 G>T	homozygous	P
DNAJC3	c.1177C>T	Homozygous	LP
ELAC2	c.680G>T	Homozygous	LP
ELAC2	c.1299G>T	Homozygous	VUS

ELAC2	c.460 T>C	homozygous	P
ETHE1	c.592dupC	Homozygous	P
FASTKD2	c.1496T>C	Homozygous	LP
FASTKD2	c.1496T>C	Homozygous	LP
FASTKD2	c.1294 C>T	homozygous	LP
FBXL4	c.292C>T	Homozygous	P
GFM1	c.1250T>C	Homozygous	
GTPBP3	c.1535 T>A	homozygous	LP
HMGCL	c.122G>A	Homozygous	P
ISCA2	c.229G>A	Homozygous	P
ISCA2	c.229G>A	Homozygous	P
ISCA2	c.229G>A	Homozygous	P
ISCA2	c.229G>A	Homozygous	P
ISCA2	c.229G>A	homozygous	P
ISCA2	c.G229A	Homozygous	P
ISCA2	c.229G>A	homozygous	P
ISCA2	c.229G>A	homozygous	P
ISCA2	c.229G>A	homozygous	P
LARS2	c.457A>C	homozygous	VUS
LRPPRC	c.1177 T>G	homozygous	VUS
MPV17	c.278A>C	Homozygous	P
MPV17	c.278A>C	Homozygous	P

MT-ND4	m.11778G>A	Heteroplasmic	P
MT-ND5	m.13513G>A	Heteroplasmic	LP
MT-ND6	m.14459 G>A	Heteroplasmic	P
MT-TL1	m.3243A>G	Heteroplasmic	P
MT-TS1	m.7471dupC	Heteroplasmic	P
MT-TS1	m.7471dupC	Heteroplasmic	P
MT-TS1	m.7471dupC	Heteroplasmic	P
NDUFS1	c.2163G>A	Homozygous	LP
NDUFS1	c.2163 G>A	homozygous	VUS
NDUFS2	c.703-11T>G	Homozygous	VUS
NDUFS8	c.460G>A	Homozygous	LP
NDUFV1	c.1312C>A	Homozygous	LP
PARS2	c.283G>A	homozygous	P
PARS2	c.283G>A	homozygous	P
PARS2	c.283G>A	homozygous	P
PARS2	c.283G>A	homozygous	P
PARS2	c.283G>A	homozygous	P
PARS2	c.283G>A	homozygous	P
PDHA1	c.419-2A>G	heterozygous	VUS
PDHA1	c.1256_1259dup	heterozygous	P
PNPLA8	c.1748_1749del	Homozygous	LP
POLG	c.2419C>T	Homozygous	P

POLG	c.925C>T	Homozygous	P
POLG	c.3286 C>T	homozygous	LP
POLG	c.911 T>G	homozygous	P
QARS	c.1058G>T	Homozygous	P
RAB3GAP2	c.1348 dup	homozygous	LP
RARS2	c.1657C>T	Homozygous	VUS
RARS2	c.1657C>T	Homozygous	VUS
SDHA	c.1753C>T	Heterozygous	LP
SERAC1	c.438del	Homozygous	P
SERAC1	c.438del	Homozygous	P
SLC25A42	c.871A>G	Homozygous	P
SLC25A42	c.871A>G	Homozygous	P
SLC25A42	c.871A>G	Homozygous	P
SLC25A42	c.871A>G	Homozygous	P
SLC25A42	c.871A>G	Homozygous	P
SLC25A42	c.871A>G	homozygous	P
SLC25A42	c.871A>G	homozygous	P
SLC25A42	c.871A>G	homozygous	LP
SLC25A42	c.871A>G	homozygous	P
SLC25A42	c.871A>G	homozygous	P
SURF1	c.823_830dup	Homozygous	P
TAZ	c.773C>A	Hemizygous	P

TMEM70	c.578_579delCA	Homozygous	P
TMEM70	c.238C>T	Homozygous	P
TMEM70	c.238C>T	Homozygous	P

P: pathogenic; LP: likely pathogenic; VUS: variant of unknown significance
